# Phenotypic Variation during Biofilm Formation: Implications for Anti-Biofilm Therapeutic Design

**DOI:** 10.3390/ma11071086

**Published:** 2018-06-26

**Authors:** Marie Beitelshees, Andrew Hill, Charles H. Jones, Blaine A. Pfeifer

**Affiliations:** 1 Department of Chemical and Biological Engineering, University at Buffalo, The State University of New York, Buffalo, NY 14260, USA; marie.beitelshees@abcombibio.com (M.B.); andrew.hill@abcombibio.com (A.H.); 2 Abcombi Biosciences Inc., 1576 Sweet Home Road, Amherst, NY 14228, USA; 3 Department of Biomedical Engineering, University at Buffalo, The State University of New York, Buffalo, New York, NY 14260, USA

**Keywords:** bacterial biofilms, commensal bacteria, bacterial phenotypes, anti-biofilm strategies, anti-adhesion, dispersion

## Abstract

Various bacterial species cycle between growth phases and biofilm formation, of which the latter facilitates persistence in inhospitable environments. These phases can be generally characterized by one or more cellular phenotype(s), each with distinct virulence factor functionality. In addition, a variety of phenotypes can often be observed within the phases themselves, which can be dependent on host conditions or the presence of nutrient and oxygen gradients within the biofilm itself (i.e., microenvironments). Currently, most anti-biofilm strategies have targeted a single phenotype; this approach has driven effective, yet incomplete, protection due to the lack of consideration of gene expression dynamics throughout the bacteria’s pathogenesis. As such, this article provides an overview of the distinct phenotypes found within each biofilm development phase and demonstrates the unique anti-biofilm solutions each phase offers. However, we conclude that a combinatorial approach must be taken to provide complete protection against biofilm forming bacterial and their resulting diseases.

## 1. Introduction

Until recently, there was little appreciation for the relationship between a bacterial phenotype and the organism’s pathogenesis. However, recent work has provided evidence that biofilms act as a primary stage of pathogenesis for up to 80% of bacterial diseases [1]. A list of common opportunistic pathogens can be found in Table 1. Interestingly, bacterial communities themselves are often asymptomatic and potentially beneficial (i.e., the microbiome). They can form on respiratory, digestive, skin, and urogenital epithelial cells, altogether colonizing a combined surface area of 300–400 m^2^ of tissue in humans [2]. While these colonies do not normally cause disease directly, disturbances in the local environment, such as viral infections or mechanical disruption, can trigger a phenotypic shift, which causes the dispersion of virulent bacteria from the biofilm. This phenotypic shift has been associated with the upregulation of virulence factors that enable the bacteria to disseminate into normally sterile regions such as the middle ear, lungs, brain, and blood, thus causing clinical conditions including otitis media, pneumonia, bacterial meningitis, and bacteremia, respectively [3,4].

Diseases caused by these dispersed bacteria are currently regulated through the use of antibiotics. However, the increasing prevalence of antibiotic resistance highlights that these measures may be short lived. Furthermore, most antibiotics are unsuccessful at clearing recalcitrant bacterial biofilms, which have the ability to partially protect normally susceptible bacteria even from high levels of antibiotics [5]. Incomplete clearance can also leave behind metabolically dormant bacteria, such as persister cells, a distinct cell type which is unaffected by antibiotics [6]. This is of particular concern as biofilms have been implicated in chronic infections, inflammation, and various genetic conditions, thus further driving the need for alternative approaches [7]. For example, *Pseudomonas aeruginosa* colonies exacerbate complications such as chronic inflammation in patients suffering from cystic fibrosis, a genetic condition of the lungs [8,9].

One shortcoming of current antimicrobial strategies is the inability to compensate for the transcriptional and phenotypic differences present in unique phases of bacterial pathogenesis. For example, commercially available pneumococcal conjugate vaccines (PCVs) target capsular polysaccharides (CPs) that are expressed during the colonizing phase of *Streptococcus pneumoniae* infection*.* However, PCVs protect against only 13 of the >95 serotypes (serotypes correspond to different versions of CPs) of *S. pneumoniae* that cause disease in humans. Therefore, these vaccines are not capable of preventing colonization of non-vaccine type (NVT) *S. pneumoniae*, which has led to a marked global increase in infectious pneumococcal disease (IPD) caused by NVT serotypes [13]. Furthermore, these vaccines are ineffective at providing protection against virulent bacteria released from the biofilm which have shed their CPs [14]. Therefore, using PCVs as the example, further protection may be offered by taking into account characteristics of other phenotypes (e.g., biofilm-detached) observed during *S. pneumoniae* pathogenesis. However, the development of such therapeutics is further complicated by phenotype variation that results from the presence of microenvironments [15,16]. It should also be noted that the presence of multiple species can affect bacterial phenotype, which has been previously covered in detail [17] and will not be discussed in this review. Finally, even if a strategy succeeds in dispersing existing biofilms, the method of dispersion may result in virulent bacteria that are phenotypically distinct from both their planktonic and biofilm counterparts [18,19]. As these bacteria could result in the subsequent biofilm formation or infectious disease, this long neglected phenotype should be accounted for in anti-biofilm strategies.

In this review, we provide an overview of the various phenotypes that exist throughout the pathogenesis of single species bacterial biofilms and highlight those studies that have made use of this knowledge to develop specific antimicrobial therapies (Table 2). However, no solution presented below is likely to become a comprehensive anti-microbial. Instead, we contend that, by understanding the phenotypes observed in each phase of biofilm development, a comprehensive picture of a target pathogen can be leveraged to inform the development of next-generation therapeutics and vaccines.

## 2. Biofilm Development Overview

Upon entering a host, bacteria are confronted with several environmental challenges such as shear forces generated by bodily fluids, host immune responses, and shifts in nutrient availability. To survive, bacteria adapt by regulating gene transcription to exhibit more favorable phenotypes for the host environment, which often culminates in biofilm development [15,38,39,40]. However, throughout this process, a diversity of factors result in many phenotypes that differ between biofilm phases and within the biofilm itself [16]. As this results in inconsistently expressed therapeutic targets and changes in metabolic state, the heterogeneity of phenotypes present a distinct challenge for developing antimicrobial treatments.

**Figure 1:**
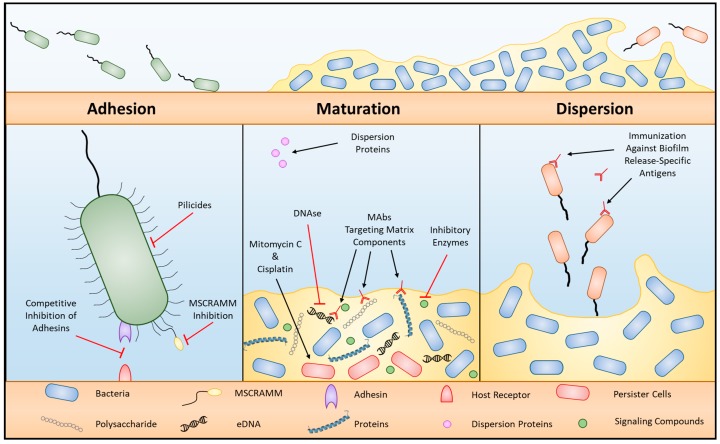
Biofilm formation and therapeutic targets. Schematic drawing of three generalized phases of biofilm formation: bacterial adhesion, biofilm maturation, and dispersion. Characteristics for each phase that represent therapeutic targets or provide opportunities for anti-biofilm strategies are highlighted.

The first step in biofilm formation involves the adherence of planktonic bacteria to anatomical surfaces, such as host epithelial cells, followed by their propagation into complex cellular communities. This process can be generalized into four stages: (1) reversible bacterial adhesion; (2) semi-irreversible attachment; (3) biofilm maturation; and (4) induced bacterial dispersion, all of which are represented by unique phenotypes [41]. Each phase offers many targets for anti-biofilm strategies (Figure 1). The first stage, initial reversible adhesion, is driven by locomotive appendages (i.e., flagella) and initiated as a response to environmental factors such as interactions with host immune cells, van der Waals and electrostatic interactions between bacterial and host surfaces, and shear forces within the body [42,43,44]. The second stage, semi-irreversible attachment, is driven by a variety of complex mechanisms that involve bacterial surface anchor proteins and macromolecule assemblies such as pili [45,46,47]. After a semi-irreversible attachment has been achieved, biofilm maturation is initiated with the production of an external matrix composed of extracellular polymeric substances (EPS) such as polysaccharides, extracellular DNA (eDNA), lipids, and proteins [48]. During or after biofilm maturation, environmental stimuli (i.e., changes in microenvironment, temperature, pH, nutrient concentration, microbial variability, and cell density) can induce the release of bacteria from the biofilm matrix (the last stage), which can then disseminate to new anatomical locations and cause disease [49].

### Quorum Sensing During Biofilm Formation

To understand the variety of bacterial phenotypes observed during biofilm formation, it is essential to have an understanding of quorum sensing (QS). In general, this process makes use of a two-component signaling transduction system (TCSTS), which consists of an intercellular response regulator, a membrane-bound histidine kinase sensor, and a signal peptide (i.e., autoinducer (AI)). When AIs accumulate to a threshold concentration, the signaling system will directly or indirectly regulate the transcription of important genes [50,51]. Both Gram-positive and Gram-negative bacteria are known to make use of QS; however, Gram-negative bacteria use luminescence (Lux) I/LuxR-type quorum sensing, which utilizes the signaling molecule acyl-hormoserine lactone (AHL), while Gram-positive pathogens encode for an oligopeptide-two-component-type quorum sensing system. A third QS pathway, distinguished by a *luxS*-encoded autoinducer 2 (AI-2), has also been identified in both Gram-negative and -positive bacteria [52] and has recently been described as the most widespread QS system identified to date [53,54].

The QS pathways have been shown to promote biofilm growth and dispersion through the regulation of essential virulence factors. For example, the expression of *luxS* in immature pneumococcal biofilms has been shown to upregulate the virulence factor *ply* and *lytA* genes [55]*.* Interestingly however, the upregulation of this pathway has exhibited an inhibitory effect on *Staphylococcus epidermidis*, thus demonstrating the complexity of QS in bacterial biofilms. Other factors for early biofilm development, such as the release of eDNA, have been linked to the expression of the cyclic-peptide-dependent accessory gene regulator (*agr*). Under certain conditions, this QS system regulates the production of autolysin E (AtlE), an enzyme that instigates the release of eDNA and facilitates surface attachment [56].

Another ubiquitous bacterial signaling system utilizing the second messenger signal, cyclic di-GMP (c-di-GMP), has been shown to control the transition from planktonic to biofilm bacteria and vice versa in multiple bacterial species [57], including *P. aeruginosa* [58,59] and *Vibrio cholerae* [60]. Unlike QS, which relies on a small number of signaling cascades to regulate transcription, c-di-GMP signaling requires multiple pathways dependent on the c-di-GMP levels to control a vast number of cellular functions [61]. This variation in molecule concentration is achieved through the use of two classes of enzymes, diguanylate cyclase (DGC) and phosphodiesterase (PDE), capable of producing or degrading c-di-GMP molecules, respectively. The resulting increase or decrease in c-di-GMP concentration is sensed by either riboswitch RNAs or c-di-GMP receptor proteins [62]. An increase in c-di-GMP levels has been linked with biofilm formation, while a decrease in concentration has been shown to result in biofilm dispersion [63]. This trend has been well defined in *P. aeruginosa*, which possesses genes encoding for five DGCs (WspR, SadC, RoeA, SiaD, and YfiN/TpbB) that help control c-di-GMP levels and regulate the transcription of genes for the transition from planktonic to biofilm bacteria and at least three PDEs (DipA (Pch), RbdA, and NbdA) that have been linked to biofilm dispersal [63]. The DGC WspR, for example, regulates the EPS production necessary for biofilm formation [64], while the PDE known as NbdA initiates biofilm dispersion upon exposure to nitric oxide [65].

## 3. Bacterial Adhesion

As mentioned above, the first critical step in biofilm formation is reversible bacterial adhesion to a surface within an anatomical location (e.g., the nasopharynx), which occurs in response to environmental stimuli, such as changes in nutrient availability and adhesion surface characteristics (i.e., surface roughness and charge) [42,66]. Other factors, such as the deposition of material by non-adhering, or detached, bacteria and the presence of naturally occurring eDNA have also been shown to increase the rate of bacterial adhesion [67,68]. During this process, planktonic bacteria are sequestered to cellular surfaces through the physical forces in the surrounding fluid or through the use of locomotive appendages. These appendages (e.g., flagella), as well as other adhesion structures (pili and curli), define the phenotypes observed during this phase, and adhesion often does not occur without them. For example, one study demonstrated that *Streptococcus pyogenes* cells lacking functional pili were unable to bind to tonsil epithelium or human keratinocytes [45].

Perhaps due to their importance in bacterial survival, both Gram-negative and -positive bacteria express a variety of pili that facilitate adhesion to host cells. The most characterized cell-surface adhesion molecule in Gram-negative bacteria is the type 1 fimbrin d-mannose specific adhesin (FimH), which facilitates bacterial binding to host glycoproteins through the use of surface-exposed terminal mannose residues [69]. Other structures of note include the P-pili, which use the PapG adhesin to bind to host oligosaccharides, and the thin amyloid fibers known as curli. The latter adhesion appendage is found in a fraction of biofilm-forming bacteria, such as clinical isolates of *Escherichia coli*, and lacks specific receptor-ligand affinity [70]. In contrast to Gram-negative bacteria, whose pili are embedded within the outer membrane, Gram-positive bacteria adhesion structures are embedded within their cell wall. Although only two types of Gram-positive pili have been identified to date (sortase assembled pili and type IV pili), these structures have demonstrated mechanisms of adhesion similar to analogous structures in Gram-negative bacteria [71].

Not long after bacteria have accumulated at cellular surfaces, cells begin to form irreversible attachments leading to the initiation of biofilm formation [41,43]. During this critical step, various bacterial genes encoding diverse and vital adhesion surface structures are upregulated, such as those responsible for the expression of surface-anchored proteins that promote adhesion to host receptors [45,46,47]. The most well studied group of surface proteins, primarily observed in Gram-positive bacteria, are microbial surface components recognizing adhesive matrix molecules (MSCRAMMs) [72]. Examples of these molecules include clumping factor B (ClfB) of *Staphylococcus aureus* [73], pneumococcal adherence and virulence factor B (PavB) of *S. pneumoniae* [74], and the M protein of *S. pyogenes* [75]. Interestingly, there is a large diversity of adhesion proteins which may have arisen as an evolutionary mechanism for evading host immune responses by interfering with the complement system and promoting inflammation. *S. aureus*, for example, expresses 24 different surface adhesion proteins that are implicated in immune evasion [76]. One of these proteins, clumping factor A (ClfA), promotes the destruction of complement factor C3b, which is recognized by receptors on host phagocytes [77,78,79]. Furthermore, expression of these proteins is dependent on location within the host, thus suggesting that host-pathogen relationships may have driven the evolution of MSCRAMMs [80,81].

### Anti-Adhesion Therapies

A better understanding of adhesion phenotypes led to antimicrobial strategies that target bacteria in the early phases of biofilm development. This is most evident in the number of strategies targeting adhesion structures, which often include competitively inhibiting bacterial adhesins and/or host receptors with the use of receptor-like molecules [82,83]. This approach has many advantages as carbohydrate-based inhibitors closely mimic host molecules and therefore are unlikely to be toxic or immunogenic [84]. For example, one study found that synthetic galabinose compounds outcompete the binding of P-fimbrated *E. coli* to galabinose-containing structures expressed on host cell surfaces [22]. In a similar fashion, several mannosides and mannose conjugates have been examined for their ability to inhibit type 1 pili-mediated adhesion, which has led to the identification of a potential therapeutic derived from the mannosidic squaric acid derivative SAMan (p-[*N*-(4-ethylamino-2,3-dioxocyclobut-1-enyl)amino]phenyl a-d-mannoside) [21]. This compound exhibited a 90% inhibition of *E. coli* attachment to human epithelial cells, making it a strong potential candidate for anti-adhesion therapy. A second popular anti-adhesion strategy is to inhibit the assembly of bacterial pili [85,86,87]. These molecules, often called pilicides, are small molecule inhibitors designed to dysregulate these adhesion appendages and prevent colonization. For example, Greene et al. engineered a molecule called pilicide ec240 which targets type 1 piliation of uropathogenic *E. coli* (UPEC) by downregulating genes in the *fim* operon, the same operon that encodes for FimH, a fimbrial adhesin associated with surface adhesion [20].

Many studies have also demonstrated the protective capabilities of vaccines composed of pili components [88,89,90,91]. Encouraging results have been observed when using pilus component proteins Spy0128 and Spy0130 from Group A Streptococci (GAS), which were able to confer >70% protection in murine models [23]. However, there is evidence that the interaction between pathogen adhesion structures and host immune response could improve bacterial adhesion. It has been found that, when recognized by the host immune system, the FimH adhesion properties are significantly enhanced by the resulting antibodies [92], suggesting that this protein may be ineffective as an antigen target in anti-bacterial vaccines.

The inhibition of MSCRAMMs has also demonstrated potential as a method to prevent bacterial adhesion. To prevent *S. aureus* and other Gram-positive infections, some studies have targeted sortase A (StrA), which enables bacterial adhesion to host cell membranes. Since this protein is not essential for bacterial growth, using StrA inhibitors creates minimal selective pressure that would lead bacteria to develop drug resistance, giving it a strong advantage over some current strategies (i.e., antibiotics) [93]. Interestingly, some promising StrA inhibitors are currently derived from biological sources such as plants and marine invertebrates [94]. For example, a compound found in many Chinese medicinal herbs, known as morin, has shown remarkable capabilities to reduce *Streptococcus mutans* biofilm formation through the inhibition of StrA [24]. Not surprisingly, there has also been interest in developing synthetic small molecule MSCRAMM inhibitors. These include pyrazolethione and pyridazinone compounds, both of which were found to have a significant negative effect on pathogen docking [25].

One cited advantage of anti-adhesion therapy is the belief that bacteria are less likely to identify an evolutionary escape mechanism, as doing so would adversely affect the pathogen’s ability to colonize the host [95]. However, many pathogens encode for more than one mechanism of adhesion which allows for host localization even when one mechanism has been blocked [95,96]. Therefore, long-term and effective anti-adhesion strategies must compensate for these diverse biological strategies.

## 4. Biofilm Maturation

As a biofilm matures, bacteria begin to shift away from adhesion phenotypes by downregulating genes controlling the expression of ahesions and pili and upregulating factors essential to survival in a bacterial community. In fact, as much as 50% of bacterial proteomes within a biofilm can be differentially expressed when compared to planktonic bacteria of the same species [97]. Multiple phenotypes arise within the biofilm during this process due to the presence of microenvironments (i.e., gradients of signaling compounds, nutrients, chemicals, oxygen, and bacterial waste) which can then govern the bacterial function and metabolic state. Therefore, cells in a mature biofilm are not only phenotypically distinct from planktonic and adhering bacteria, they also form phenotypically distinct regions within an individual biofilm [16,98]. This heterogeneity has also been shown to lead to a division of labor in which cells perform specialized tasks to benefit the cellular community [99]. Common examples found within mature biofilms include biofilm matrix producers and persister cells, both of which provide unique challenges as well as promising targets for anti-biofilm strategies.

### 4.1. Extracellular Matrix Producers

The matrix producing cells within bacterial biofilms are responsible for the production of EPSs (i.e., polysaccharides, nucleic acids, lipids, and proteins) [100]. In some bacterial biofilms, such as *Bacillus subtilis*, this cell type is located primarily in the core of the biofilm in order to maintain its structure and rigidity [98]. Beyond providing structure, the EPS produced enhances biofilm formation by facilitating cell-cell communication and acting as a shield against numerous environmental hazards (i.e., antibiotics). They also serve as an external digestion system by breaking down lysed bacterial cells into nutrients that can be recycled to cells within the biofilm [48]. The most common components of the biofilm matrix are exopolysaccharides, eDNA, and proteins; however, the composition of EPSs is highly dependent on bacterial species and host conditions.

Matrix producing bacteria have been shown to excrete exopolysaccharides, the type of which can also impact bacterial phenotype. For example, during early biofilm formation, *P. aeruginosa* expresses a non-mucoid phenotype. These bacteria primarily produce Pel and Psl as structural exopolysaccharides, which have been found to play roles in increasing biofilm cell density and initial cell attachment, respectively [101,102,103]. However, over time, this bacterium can switch to the mucoid phenotype, which poses particular problems for cystic fibrosis patients. This switch, due to genetic mutations of the anti-sigma factor MucA, has been attributed to the overexpression of the exopolysaccharide alginate which enhances resistance to antibiotics and host immune cells as well as provides matrix structure [104,105]. As with all EPSs, the type of exopolysaccharide produced varies between bacterial species. However, there is one that is conserved throughout many microbial species, encompassing both Gram-positive and –negative bacteria: poly-β(1-6)-*N*-acetylglucosamine (PNAG) [102,106]. In bacteria such as *S. aureus*, this polysaccharide provides the main functional component of intracellular adhesion [107].

A second category of EPS, eDNA, which can facilitate adhesion during the early phase of biofilm development, has recently been shown to provide structural support within the biofilm matrix. However, the structural contribution of eDNA varies between bacterial species. For example, while eDNA is only a minor component in *S. epidermidis* biofilms, it is a major structural component in *P. aeruginosa* biofilms. To demonstrate this, an absence of eDNA in *P. aeruginosa* biofilms has been shown to negatively impacts 3-dimensional (3D) biofilm development without impairing individual cell growth, further establishing a role for eDNA in biofilm structure [108]. To build upon this theory, studies have hypothesized that eDNA may be used as scaffolding for the initial 3D structure as the integrity of mature biofilms is only minimally impacted by DNase, an enzyme that can completely dissolve biofilms in their early phases [108]. eDNA is also capable of interacting with other EPSs, such as Psl and Pel, which results in strong biofilm “skeletons” capable of reducing the effectiveness of DNase [109]. The presence of eDNA within the matrix could be the result of passive release from dead cells, active release from physiologically active cells, or bacteriophage infection spurring release [110]. For example, in some cases, eDNA appears to originate from a small subpopulation of autolytic cells, the activation of which are controlled by the *cidA* gene in *S. aureus* [111]. This gene, in turn, is regulated by the LytSR two-component regulatory system [112]. In contrast, nontypeable *Haemophilus influenzae* (NTHI) has recently been shown to secrete chromosomal DNA during biofilm maturation via a two-pore system. It has been shown that this bacteria uses an inner-membrane pore (TraCG) to transport eDNA to the periplasm and ComE to secrete the EPS to the biofilm matrix [113].

Matrix-producing bacteria also excrete matrix proteins. While EPS and eDNA have understood roles in biofilm structure, little is known about the roles proteins play in the biofilm matrix and information regarding the identities of these proteins is scarce. A recent proteomic study sought to characterize the proteins found in the *P. aeruginosa* matrix and found that they were composed largely of outer membrane proteins, secreted proteins, and the contents of lysed cells, including many well-characterized virulence factors [114]. One such protein, cyclic diguanylate-regulated TPS partner A (CdrA), was shown to link cells to the Psl exopolysaccharide, thus reinforcing the biofilm matrix [115]. In addition, DNA binding proteins, like DNA-binding protein HU (PA1804), are also common contributors to EPSs and are thought to alter gene transcription. However, despite their DNA biding properties, it is not yet known if they interact with eDNA [116].

### 4.2. Persister Cells

During biofilm maturation, microenvironments form that have distinct impacts on bacterial phenotype. These regions can be divided into three generalized categories: (1) an oxygen and substrate rich zone on or near the surface of the biofilm; (2) an intermediate substrate rich and oxygen depleted zone in which cells depend heavily on fermentation; (3) and a substrate and oxygen depleted zone consisting of metabolically dormant near the adhesion surface [16].

Most matrix-producing cells can be found within the first two zones. However, within substrate and oxygen depleted zones of a biofilm a divergent subpopulation of persister cells can be found, which has been a hindrance to the development of effective antimicrobial strategies. These cells exist as a small portion of biofilms that are tolerant to antibiotics while remaining protected from the host immune system. As such, the susceptible cells are killed during an antibiotic regimen, leaving the persister cells to repopulate the biofilms. This not only renders the antimicrobial strategy ineffectual, but can also lead to chronic infections [6]. Like antibiotic resistant bacteria, which obtain resistance through genetic mutations, persister cells are notoriously difficult to treat. However, in contrast to antibiotic resistant cells, persister cells obtain tolerance via transition to a metabolically dormant state that no longer expresses most antimicrobial targets, without undergoing any genetic modifications [117,118].

### 4.3. Anti-Biofilm Strategies

**Figure 2:**
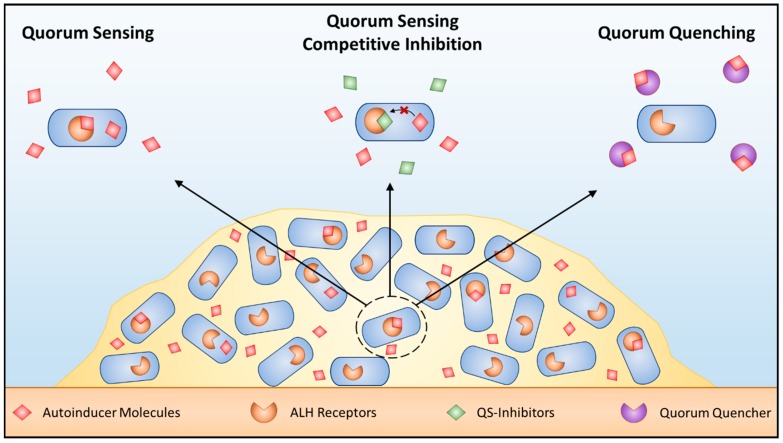
Targeting quorum sensing. Schematic of QS in bacteria as well as methods to block this signaling mechanism. AHL dependent QS within biofilms (left) can be blocked using competitive QS inhibition that outcompete AHL for AHL receptors (middle) or quorum quenching enzymes that inactivate AHL signals (right).

As bacterial sensing (i.e., QS and c-di-GMP signaling) drives phenotypic variation during biofilm maturation, it is unsurprising that inhibition of these processes can provide effective antimicrobial strategies. A number of methods have been developed that fall under two categories: QS inhibiters and quorum quenchers (QQs) (Figure 2). The first method, QS inhibition, aims to block QS by introducing small-molecule analogs that outcompete signal molecules [119]. Using this strategy, it is possible to alter cellular phenotypes expressed within mature biofilms. For example, the *agr* signaling pathway, despite being implicated in initial cell adhesion under certain conditions, is also capable of inhibiting important biofilm matrix proteins (fibronectin-binding proteins (FnBPs) and Protein A) produced by *S. aureus* [56,120]. To exploit this natural system as an anti-biofilm strategy, it is possible to activate the *arg* signaling system through the addition of autoinducing peptides (AIPs). When combined with serine proteases, AIPs were highly effective at dispersing established, mature biofilms; however, this strategy would be ineffective at targeting *agr* deficient strains [121]. Conversely, quorum quenchers shut down QS via enzyme inhibitors which can be classified as: (1) lactonases; (2) acylases; and (3) oxidoreductases [122]. Most QQ enzymes identified to date fall under the category of lactonases, due to their ability to degrade AHL molecules [123,124,125]. One such enzyme, *Sso*Pox-W263I, was capable of decreasing the virulence of clinical isolates of *P. aeruginosa* from diabetic foot ulcers by disrupting QS and reducing biofilm formation [26]. Finally, many strategies have attempted to disrupt c-di-GMP signaling due to its large role in regulating the bacterial phenotypes that produce exopolysaccharides and matrix proteins. For example, high levels of c-di-GMP have been linked to increased production of the polysaccharides Pel and Psl and the protein CdrA in *P. aeruginosa* [115,126]. Therefore, degradation of this compound presents an interesting anti-biofilm solution. One PDE of interest, the regulatory enzyme BsmR not only degrades c-di-GMP, but also upregulates genes associated with biofilm dispersal, making it a strong antimicrobial candidate [27].

An additional strategy to modify phenotypes within mature biofilms is the transcriptional alteration of essential enzymes. This is possible due to technologies such as CRISPR (clustered regularly interspaced short palindromic repeats), which allows for the alteration of any target gene [127]. One target of particular interest is LuxS, the enzyme that synthesizes AI-2, as it has been shown to influence matrix producing phenotypes. For example, it regulates the production of eDNA through the activation of LytA-dependent autolysis activity in *S. pneumoniae* [128]. As eDNA provides structure to biofilms, inhibition of LuxS could prevent biofilm formation or weaken existing communities. Interestingly, recent studies demonstrated that CRISPR inhibition (i.e., CRISPRi) could knockdown *luxS* which, in turn, prevented metabolically active *E. coli* from developing biofilms, likely due to the prevention of EPS production through AI-2 inhibition [28,29]. These studies appear to be the first attempts to utilize CRISPR in an effort to prevent or eliminate biofilms; however, it may be possible to knockout other genes essential to biofilm maturation.

Instead of targeting various phenotypes, it is also possible to target cellular products in order to weaken or disperse mature biofilms [129,130]. The introduction of dispersion proteins, such as dispersin B of *Aggregatibacter actinomycetemcomitans,* has the ability to inhibit initial biofilm formation, detach existing colonies, and compromise the physical integrity of the matrix in staphylococcal biofilms by attaching to the exopolysaccharide PIA [30,48,49,131,132]. The enzyme DNase may also be effective at dispersing early-staged biofilms and increasing their susceptibility to antibiotics by targeting eDNA within the matrix, thus leading many researchers to analyze its potential as an anti-biofilm therapy [30,133,134]. This strategy has culminated in Genentech’s Pulmozyme^®^, a recombinant human DNase I that targets *P. aeruginosa* infections of cystic fibrosis patients. Antibody-based therapies (i.e., monoclonal antibodies) may also be able to eliminate mature biofilms [135,136,137]. One antigen of interest is PNAG, an exopolysaccharide that is conserved in many bacterial species, and antibodies targeting this antigen have been shown to prevent and eliminate bacterial biofilms [138]. This success has led to the development of an anti-PNAG monoclonal antibody which has completed a Phase I clinical trial [32,139].

While the strategies detailed above may be effective at targeting the matrix producing phenotypes, resulting dispersion may leave persister cells behind which can repopulate the biofilm. As these cells are metabolically dormant, anti-microbial compounds targeting persister cells must be capable of entering cells without the need for active transport. In addition, their mechanism of action must require no innate cellular machinery [140]. Such compounds exist and include the chemotherapeutic agents mitomycin C and cisplatin [33,141]. Additionally, studies have shown that persister cells can be reawakened, after which they can be targeted with traditional antibiotics. This has been done with *cis*-2-decenoic acid, which revitalized protein synthesis within the previously dormant cells [35].

## 5. Dispersion

During or after biofilm maturation, a subpopulation of bacteria can disperse from the biofilm matrix to colonize other regions of the host. This population represents a phenotype distinct from both planktonic and biofilm bacteria [141,142]. During this phase, genes for motility and virulence are upregulated in response to environmental cues (i.e., cell density, febrile conditions, bacteriophage infection, changes in nutrient availability) [4,143]. Dispersion can occur as either single motile cells or as multicellular aggregates.

Interestingly, the dispersed single motile cell phenotype has a greater capacity to develop biofilms when compared with planktonic bacteria [19]. However, high density aggregates, which retain similar phenotypes to biofilm bacteria, surpass both planktonic and single dispersed cells in the ability to form biofilms, making them important to disease pathogenesis [144]. As further evidence that biofilm detachment plays a large role in bacterial diseases, there is also increasing evidence that certain methods of dispersal can result in the release bacteria more virulent than their planktonic counterparts [4]. For example, fever-induced biofilm release of pneumococci has been shown to result in bacterial dissemination to the lungs and blood to a greater degree than observed for planktonic bacteria in murine models [36], and it is now apparent that the biofilm colonization phase is essential for disease progression. As the end goal of most anti-biofilm strategies is biofilm dissemination, there is a possibility of inadvertently activating this phenotype; therefore, secondary measures (e.g., antibiotics, vaccines) should be taken to prevent further spread of disease.

In addition, many biofilms are asymptomatic and potentially beneficial to the host (i.e., microbiota) [145]. Therefore, a better understanding of the biofilm-released phase offers the potential for innovative strategies targeting opportunistic pathogens while leaving potentially beneficial bacteria in place. This idea was put into practice during the recent transcriptome analysis of planktonic, biofilm-forming, and biofilm-released cells which identified protein antigens specific to the biofilm-release phase [4]. Two of these proteins, alpha-glycerophosphate oxidase (GlpO) and the bacterocin PncO, were found to be homologous throughout *S. pneumoniae* strains and offered complete protection against this virulent phenotype in murine models when combined into a single vaccine [36,37]. To our knowledge, this is the first strategy to specifically target biofilm-detached bacteria, while leaving the biofilm intact.

## 6. Conclusions

To develop effective therapeutic strategies against biofilm-forming bacteria, it is essential to understand the phenotypic diversity that is observed within these biofilms. These differences pose many challenges to researchers targeting these colonizing bacteria. However, with a better understanding comes the potential for effective treatment and vaccination strategies such as those mentioned above. While each strategy mentioned in this review has its strengths, few have ever demonstrated the coverage needed to provide full protection against their target pathogen. This is due to the fact that many strategies focus only on one aspect of bacterial diversity. Therefore, combining strategies may provide universal protection against diseases caused by biofilm forming bacteria. For example, as biofilm dispersion therapies may result in virulent biofilm-released bacteria, secondary treatments may be required to prevent the further spread of disease. In summary, this review presents evidence that taking phenotypic differences into account will enable the development of widely effective anti-infective solutions.

## Figures and Tables

**Table 1:** List of colonizing bacterial pathogens.

Pathogen	Disease	Colonization Site	**Incidence Rate ^A^**	**Fatality Rate ^A^**
*Streptococcus pneumoniae*	Pneumonia	Nasopharynx	9.5 ^A^	1.14 ^A^
*Staphylococcus aureus* (MRSA)	Skin infection	Nasopharynx, Skin	22.72 ^A^	2.88 ^A^
Group A Streptococcus	Strep throat	Pharynx	5.8 ^A^	0.58 ^A^
*Haemophilus influenzae*	Bacteremia	Nasopharynx	1.99 ^A^	0.29 ^A^
*Neisseria meningitidis*	Meningitis	Nasopharynx	0.12 ^A^	0.01 ^A^
Legionellosis	Atypical pneumonia	Lungs	1.42 ^A^	0.1 ^A^
*Moraxella catarrhalis*	Otitis media	Nasopharynx	N/A	0 ^A^
Group B Streptococcus	Septicemia	Gastrointestinal tract	9.6 ^A^	0.53 ^A^
*Porphyromonas gingivalis*	Periodontal disease	Oral Cavity	9.24 ^B^	-
*Escherichia coli* *Pseudomonas aeruginosa* *Klebsiella pneumoniae*	Catheter- Associated Urinary Tract Infection (CAUTI)	Bladder Catheter	3.3 ^C^	17.3 ^C^
	Ventilator-Associated Pneumonia (VAP)	Ventilator	3.3 ^C^	15.2 ^C^
*Escherichia coli* *Staphylococcus aureus* *Pseudomonas aeruginosa*	Prosthetic Joint Infections (PJI)	Prosthetic Joints (e.g., hip, knee)	1.5򢀓2.5 ^D^	2.5 ^D^

^A^ Incidence or Fatality rate of disease per 100,000 obtained from CDC’s Active Bacterial Core Surveillance program. ^B^ [10]. ^C^ Incidence rate per 1000 Catheter/Ventilator-days; Fatality rate per 100 CATUI/VAP cases [11]. ^D^ Incidence rate per 100 arthroplasties; Fatality rate per 100 PJI cases [12].

**Table 2:** Summary of anti-biofilm strategies by biofilm phase.

Target	Bacteria	Anti-Microbial Strategy	Reference
***Anti-Adhesion Phenotype Strategies***			
Type I Pili	*Escherichia coli*	Pilicide ec240	[20]
		SAMan	[21]
P-fimbrate	*Escherichia coli*	Synthetic galabinose	[22]
Spy0128 and Spy0130	Group A Streptococcus	Vaccination	[23]
StrA	*Streptococcus mutans*	Morin	[24]
StrA	*Staphylococcus aureus*	pyrazolethione and pyridazinone	[25]
***Anti-Biofilm Phenotype Strategies***			
AHL Molecules	*Pseudomonas aeruginosa*	SsoPox-W263I	[26]
c-di-GMP	*Stenotrophomonas maltophilia*	BsmR	[27]
LuxS	*Streptococcus pneumoniae*	CRISPR	[28,29]
PIA	*Staphylococcus*	dispersin B	[30]
eDNA	*Pseudomonas aeruginosa*	DNAse I (Pulmozyme^®^)	[31]
PNAG	*S. aureus*	Monoclonal Antibody	[32]
Persister Cells	*Escherichia coli*	Mitomycin C	[33]
Persister Cells	*Pseudomonas aeruginosa*	Cisplatin	[34]
Persister Cells	*Pseudomonas aeruginosa* *Escherichia coli*	*cis*-2-Decenoic Acid	[35]
***Anti-Dispersed Bacteria Phenotype Strategies***			
GlpO	*Streptococcus pneumoniae*	Vaccine with GlpO Antigen	[36,37]
PncO	*Streptococcus pneumoniae*	Vaccine with PncO Antigen	[36,37]
